# Association of Age With Treatment-Related Adverse Events and Survival in Patients With Metastatic Colorectal Cancer

**DOI:** 10.1001/jamanetworkopen.2023.20035

**Published:** 2023-06-26

**Authors:** Lingbin Meng, Ram Thapa, Maria G. Delgado, Maria F. Gomez, Rui Ji, Todd C. Knepper, Joleen M. Hubbard, Xuefeng Wang, Jennifer B. Permuth, Richard D. Kim, Damian A. Laber, Hao Xie

**Affiliations:** 1 Department of Hematology and Oncology, H. Lee Moffitt Cancer Center & Research Institute, Tampa; 2 Division of Medical Oncology, The Ohio State University College of Medicine, Columbus; 3 Department of Biostatistics and Bioinformatics, H. Lee Moffitt Cancer Center & Research Institute, Tampa, Florida; 4 Department of Cancer Epidemiology, H. Lee Moffitt Cancer Center & Research Institute, Tampa, Florida; 5 Department of Personalized Cancer Medicine, H. Lee Moffitt Cancer Center & Research Institute, Tampa, Florida; 6 Division of Medical Oncology, Mayo Clinic, Rochester, Minnesota; 7 Department of Gastrointestinal Oncology, H. Lee Moffitt Cancer Center & Research Institute, Tampa, Florida

## Abstract

**Importance:**

While the incidence of early-onset metastatic colorectal cancer (mCRC) has been increasing, studies on the age-related disparity in this group of patients are limited.

**Objective:**

To evaluate the association of age with treatment-related adverse events and survival in patients with mCRC and explore the potential underlying factors.

**Design, Setting, and Participants:**

This cohort study included 1959 individuals. Individual data on 1223 patients with mCRC who received first-line fluorouracil and oxaliplatin therapy in 3 clinical trials, and clinical and genomic data of 736 patients with mCRC from Moffitt Cancer Center were used to assess genomic alterations and serve as an external validation cohort. All statistical analyses were conducted from October 1, 2021, through November 12, 2022.

**Exposures:**

Metastatic colorectal cancer.

**Main Outcomes and Measures:**

Survival outcomes and treatment-related adverse events were compared among patients in 3 age groups: younger than 50 (early onset), 50 to 65, and older than 65 years.

**Results:**

In the total population of 1959 individuals, 1145 (58.4%) were men. Among 1223 patients from previous clinical trials, 179 (14.6%) in the younger than 50 years group, 582 (47.6%) in the 50 to 65 years group, and 462 (37.8%) in the older than 65 years group had similar baseline characteristics except for sex and race. The younger than 50 years group had significantly shorter progression-free survival (PFS) (hazard ratio [HR], 1.46; 95% CI, 1.22-1.76; *P* < .001) and overall survival (OS) (HR, 1.48; 95% CI, 1.19-1.84; *P* < .001) compared with the 50 to 65 years group after adjustment for sex, race, and performance status. Significantly shorter OS in the younger than 50 years group was confirmed in the Moffitt cohort. The younger than 50 years group had a significantly higher incidence of nausea and vomiting (69.3% vs 57.6% [50-65 years] vs 60.4% [>65 years]; *P* = .02), severe abdominal pain (8.4% vs 3.4% vs 3.5%; *P* = .02), severe anemia (6.1% vs 1.0% vs 1.5%; *P* < .001), and severe rash (2.8% vs 1.2% vs 0.4% *P* = .047). The younger than 50 years group also had earlier onset of nausea and vomiting (1.0 vs 2.1 vs 2.6 weeks; *P* = .01), mucositis (3.6 vs 5.1 vs 5.7 weeks; *P* = .05), and neutropenia (8.0 vs 9.4 vs 8.4 weeks; *P* = .04), and shorter duration of mucositis (0.6 vs 0.9 vs 1.0 weeks; *P* = .006). In the younger than 50 years group, severe abdominal pain and severe liver toxic effects were associated with shorter survival. The Moffitt genomic data showed that the younger than 50 years group had a higher prevalence of *CTNNB1* mutation (6.6% vs 3.1% vs 2.3%; *P* = .047), *ERBB2* amplification (5.1% vs 0.6% vs 2.3%; *P* = .005), and *CREBBP* mutation (3.1% vs 0.9% vs 0.5%; *P* = .05), but lower prevalence of *BRAF* mutation (7.7% vs 8.5% vs 16.7%; *P* = .002).

**Conclusions and Relevance:**

In this cohort study of 1959 patients, those with early-onset mCRC showed worse survival outcomes and unique adverse event patterns, which could be partially attributed to distinct genomic profiles. These findings may inform individualized management approaches in patients with early-onset mCRC.

## Key Points

**Question:**

Is there any difference in treatment-related adverse events and outcomes between patients with early-onset (age <50 years) and older patients with metastatic colorectal cancer (mCRC)?

**Findings:**

In this cohort study of 1223 patients with mCRC from 3 clinical trials, early-onset mCRC had significantly worse survival and displayed unique patterns of treatment-related adverse events. Analysis in a separate Moffitt Cancer Center cohort of 736 patients noted worse overall survival in early-onset mCRC and showed distinct genomic profiles in this population, which may partially explain the disparity.

**Meaning:**

These findings might have utility for an individualized approach to chemotherapy, counseling, and management of treatment-related adverse events in patients with early-onset mCRC.

## Teaser

This cohort study compares survival outcomes and adverse event patterns among patients with early-onset metastatic colorectal cancer with those of older patients.

## Introduction

Colorectal cancer (CRC) is the third most common cancer and the second leading cause of cancer-related death worldwide.^1^ Despite the decrease of overall incidence and mortality of CRC as a result of nationwide-adopted screening programs,^2,3,4^ the incidence of the early-onset CRC (EO-CRC) in patients diagnosed with CRC before age 50 years has been increasing by 2% annually since the 1990s.^5,6^ It is projected that by 2030 10.9% of all colon cancers and 22.9% of all rectal cancers will be diagnosed as EO-CRC.^7,8^

Neither the etiology of increased incidence nor the distinct biology of EO-CRC compared with its older counterparts is clearly revealed. For example, inherited cancer syndromes, such as Lynch syndrome, may be more prevalent in patients with EO-CRC. However, only 5% to 7% of those with EO-CRC carry deleterious germline mutations of mismatch repair genes.^9,10^ Previous studies examining the genomic profile of EO-CRC showed few molecular differences between EO-CRC and its older counterparts.^11^ Even the few molecular differences reported were often not confirmed in other studies likely due to selection bias in different patient cohorts.^8,12,13^ Some studies investigated nongenetic risk factors for CRC, such as low physical activity, excess alcohol consumption, smoking, and obesity, but the results were neither conclusive nor adequate explanations of the increased incidence of EO-CRC.^14,15,16^

The increased incidence of EO-CRC poses unique challenges to the management of treatment in younger patients, especially with regard to the prognosis and treatment-related adverse events.^17^ In current clinical practice, patients with EO-CRC receive similar, if not more aggressive, therapies in the advanced disease setting compared with their older counterparts. Fluoropyrimidine-based combination chemotherapy plus biologic agents remains the most common first-line therapy for patients with metastatic colorectal cancer (mCRC).^18^ Triplet chemotherapy combination is an alternative for patients who are younger and have more aggressive disease and good performance status, offering a better outcome at the expense of worse toxic effects.^19^ Although chemotherapy in combination with biologic agents^20,21^ showed comparable benefit in patients with advanced CRC of all ages,^22,23^ it is unclear whether there is disparity in treatment-related adverse events and outcome from advanced CRC between EO and its older counterparts. Previous studies suggested an increased incidence of some specific toxic effects, such as nausea and vomiting in EO-mCRC, but the data were largely limited in the adjuvant setting and findings across studies were not always in agreement.^24,25,26,27,28^ Similarly, some studies reported a poorer survival in patients with advanced EO-CRC,^24,25,29^ whereas others observed a similar survival outcome between patients with EO-mCRC and those with average onset.^26,27,28,30,31^ These discrepancies might be due to selection bias and/or the inclusion of older patients (age >65 years) in the average-onset group for comparison.

To address this literature gap, we used individual patient data from 3 clinical trials in which patients with mCRC received first-line fluorouracil and oxaliplatin (FOLFOX) therapy and a contemporary patient cohort to compare the differences of treatment outcome and adverse events among 3 different age groups of patients with mCRC.

## Methods

We used individual patient data from 3 multicenter randomized phase 3 clinical trials in the Project Data Sphere, which were divided into study 1 (NCT00272051, NCT 00305188) and study 2 (NCT00364013). Study 1 assessed the efficacy of xaliproden in reducing (NCT00272051) or preventing (NCT 00305188) the neurotoxic effects of FOLFOX as first-line treatment for patients with mCRC. Study 2 evaluated the efficacy of panitumumab combined with FOLFOX as first-line therapy for patients with mCRC. Only patient data from the control (FOLFOX only) arms of the 3 clinical trials were combined and used in our study. Study design, inclusion and exclusion criteria, interventions, end points, adverse events, and outcomes of the 3 clinical trials have been previously reported^23,32,33,34,35^ and are briefly described in the eMethods in Supplement 1. Given these patients were treated in a clinical trial setting before 2010, we included a contemporary patient population as an external validation cohort for overall survival (OS). Patients of this cohort were identified from the prospectively maintained Moffitt Clinical Genomic Action Committee Database and CARIS clinical database. These include patients diagnosed with mCRC and treated at Moffitt Cancer Center (MCC) from 2006 to 2022 and have available clinical and next-generation sequencing (NGS) data. Clinical NGS data were provided by common commercially available platforms, including FoundationOne, FoundationOne ACT, FoundationOne CDx, CARIS, and Guardant360, along with an in-house NGS assay called Moffitt STAR. These platforms have been described in depth elsewhere.^36,37,38^ The patient data from the 3 clinical trials were deidentified and sourced from a central, password-protected database managed by Project Data Sphere. Access to these data for research purposes was granted by the Project Data Sphere scientific committee. The MCC Scientific Review Committee and the Institutional Review Board granted approval for the collection and analysis of data from the Moffitt Clinical Genomic Database and waived the requirement for informed consent from study participants because of the use of deidentified data. Racial information used in our research was obtained from these databases, with data including Asian, Black, White, unknown, and other. Data on races other than White were combined because of very small sample sizes. This study adhered to the reporting requirements established by the Strengthening the Reporting of Observational Studies in Epidemiology (STROBE) reporting guideline.

Overall survival was defined as the time from randomization to death. Participants who were alive at the analysis data cutoff were censored at their last contact date. Progression-free survival (PFS) was defined as the time from randomization to date of disease progression assessed radiographically per the Response Evaluation Criteria in Solid Tumors 1.0. Safety end points included incidence of any grade and grade 3 to 5 (severe) treatment-related adverse events according to the National Cancer Institute Common Terminology Criteria for Adverse Events, version 3.0, the time of their onset, and their duration. Analyses of the differences of genetic alterations among 3 age groups were exploratory.

### Statistical Analysis

Our statistical analysis was performed using R Statistical Software, version 4.1.2 (R Foundation for Statistical Computing) along with the survival package 3.2-13. The χ^2^ test and Fisher exact test were used to test the distribution of baseline characteristics, adverse events, and NGS genomic features in the 3 groups. Continuous variables were compared with the Kruskal-Wallis test and χ^2^ or Fisher exact test were performed for categorical variables. Overall survival and PFS were evaluated according to the Kaplan-Meier method and compared using the log-rank test. A Cox proportional hazards regression model was used to determine the association of adverse events with OS and PFS. *P* values in this study were 2-sided, and *P* < .05 was considered statistically significant. Pairwise comparisons adjusting for multiple testing were performed using the Benjamini and Hochberg method.

## Results

### Demographic and Clinical Characteristics

Among all 1959 patients (1145 [58.4%] men; 814 [41.6%] women) included, 1223 were from study 1 and study 2 for analyses of survival outcomes and treatment-related adverse events. A total of 736 patients were from the Moffitt cohort for assessment of OS and tumor genetic alterations. As presented in the Table, the Moffitt cohort included a significantly higher rate of patients with EO-mCRC (26.6%) compared with study 1 (15.2%) and study 2 (13.7%) (*P* < .001), but a lower prevalence of White patients (83.3%) vs those of other races and ethnicities (16.7%) (*P* < .001). Of the patients from study 1 and study 2, 179 (14.6%) were younger than 50 years, 582 (47.6%) were aged 50 to 65 years, and 462 (37.8%) were older than 65 years at the time of stage IV CRC diagnosis (eTable 1 in Supplement 1). There were significantly more women younger than 50 years in contrast to more men older than 65 years. Fewer White patients and more individuals of other races and ethnicities were represented in the age less than 50 years group. No significant difference of Eastern Cooperative Oncology Group (ECOG) performance status was observed among the 3 age groups (eTable 1 in Supplement 1).

**Table.** Demographic and Clinical Characteristics of Patients With Metastatic Colorectal Cancer From the 3 Cohorts

Variable	No. (%)^a^				*P* value
	Overall (N = 1959)	Study 1 (n = 756)	Study 2 (n = 467)	Moffitt (n = 736)	
Age, y					
<50	375 (19.1)	115 (15.2)	64 (13.7)	196 (26.6)	<.001
50-65	901 (46.0)	358 (47.4)	224 (48.0)	319 (43.3)	
>65	683 (34.9)	283 (37.4)	179 (38.3)	221 (30.0)	
Sex					
Female	814 (41.6)	304 (40.2)	182 (39.0)	328 (44.6)	.10
Male	1145 (58.4)	452 (59.8)	285 (61.0)	408 (55.4)	
Race^b^					
White	1742 (89.5)	680 (89.9)	460 (98.5)	602 (83.3)	<.001
Other^c^	204 (10.5)	76 (10.1)	7 (1.5)	121 (16.7)	
ECOG^d^					
0/1	1182 (96.9)	739 (98.0)	443 (95.1)	NA	.007
2	38 (3.1)	15 (2.0)	23 (4.9)	NA	

Abbreviations: ECOG, Eastern Cooperative Oncology Group; NA, not available.

^a^ Data incomplete in some categories.

^b^ Race (n = 1946) calculated on total number within that category vs overall population.

^c^ Other race included Asian, Black, and other. Data on races other than White were combined because of very small sample sizes.

^d^ ECOG score (n = 1220) calculated on total number within that category vs overall population.

### Age-Related Disparity of Survival Outcome

**Figure 1.**
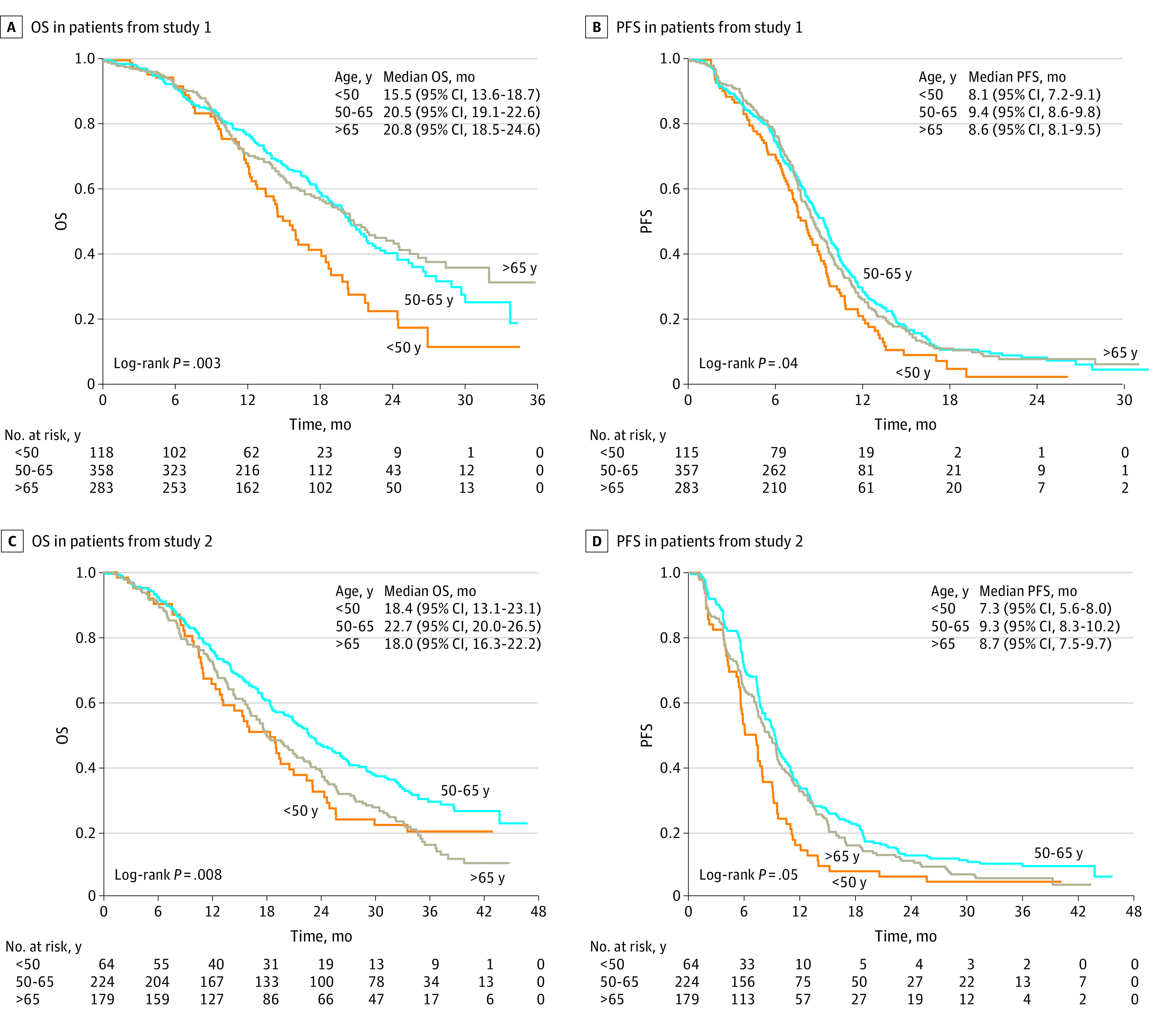
Survival Outcome of Patients With Metastatic Colorectal Cancer Stratified by Age Group OS indicates overall survival; PFS, progression-free survival.

In study 1, patients younger than 50 years had shorter median OS compared with those aged 50 to 65 and older than 65 years (15.5 vs 20.5 vs 20.8 months; *P* = .003) (Figure 1A). Patients younger than 50 years also had shorter median PFS than that of the other 2 age groups (8.1 vs 9.4 vs 8.6 months; *P* = .004) (Figure 1B). Similar findings were observed in study 2, as shown in Figure 1C (median OS, 18.4 vs 22.7 vs 18.0 months; *P* = .008) and in Figure 1D (median PFS, 7.3 vs 9.3 vs 8.7 months; *P* = .005). In univariate survival analysis, age younger than 50 years (<50 vs 50-65 years: hazard ratio [HR], 1.50; 95% CI, 1.21-1.85; *P* < .001) and poor ECOG performance status (2 vs 0/1: HR, 2.49; 95% CI, 1.75-3.54; *P* < .001) were identified as poor prognostic factors for OS in both study 1 and study 2 combined. Age younger than 50 years was noted in multivariable analysis to be an independent factor for worse OS (age <50 vs 50-65 years: HR, 1.48; 95% CI, 1.19-1.84; *P* < .001) after adjustment for sex, race, and ECOG performance status. Age younger than 50 years was similarly found to be an independent factor for poor PFS (age <50 vs 50-65 years: HR, 1.46; 95% CI, 1.22-1.76; *P* < .001), as reported in eTable 2 in Supplement 1.

### Age-Related Disparity of Treatment-Related Adverse Event Pattern

**Figure 2.**
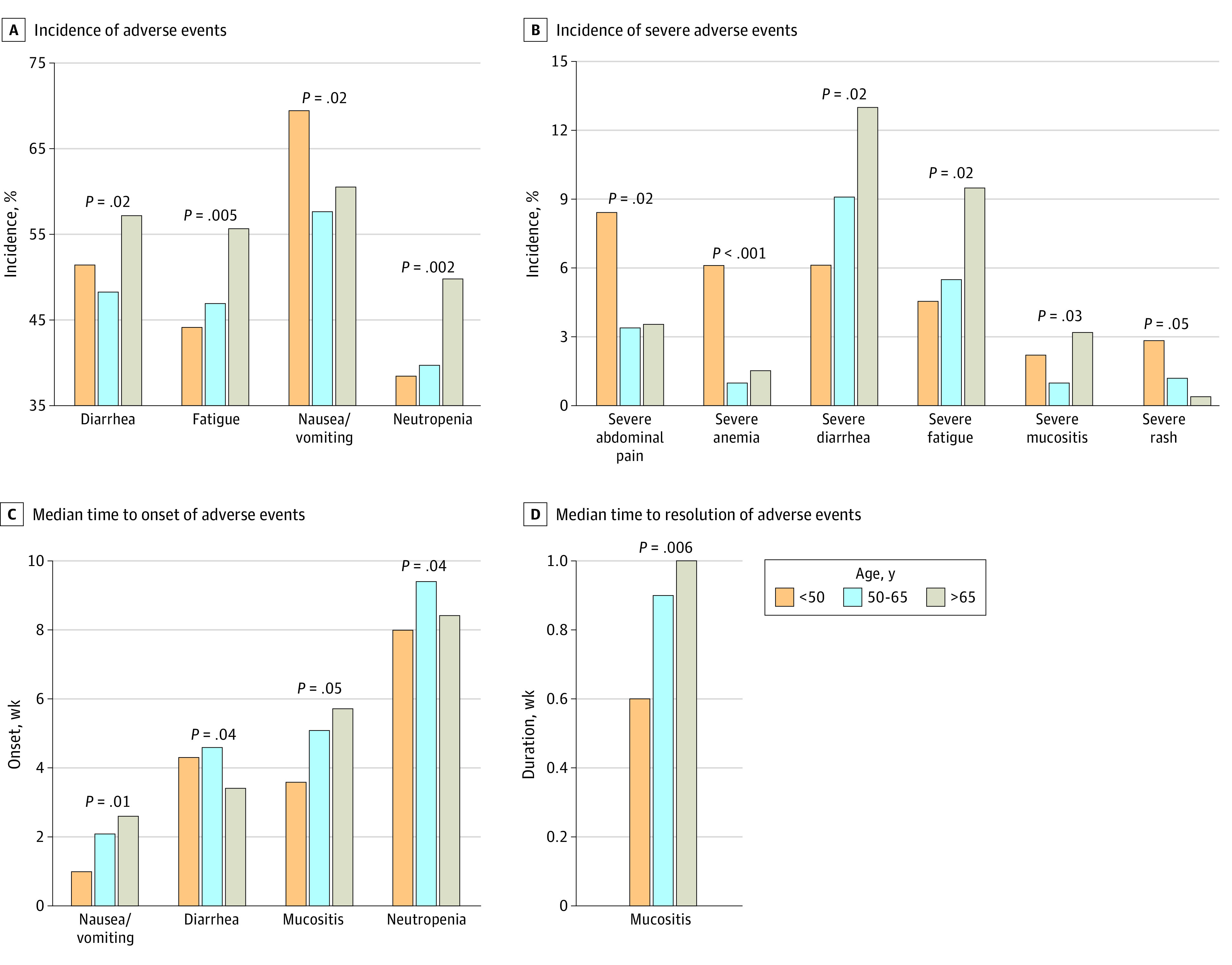
Adverse Event Pattern of Patients With Early-Onset Metastatic Colorectal Cancer in Study 1 and Study 2 Incidence of adverse events (A), incidence of severe (grade 3-5) adverse events (B), median time to onset of adverse events (C), and median time to resolution of adverse events (D) for each age group with statistically significant differences.

Examination of treatment-related adverse events of all patients from study 1 and study 2 combined revealed a unique pattern for patients younger than 50 years (Figure 2). As shown in Figure 2A, B and eTable 3 in Supplement 1, compared with the study 1 and study 2 age groups, patients younger than 50 years had a higher incidence of nausea and vomiting (69.3% vs 57.6% 60.4%; *P* = .02), severe abdominal pain (8.4% vs 3.4% vs 3.5%; *P* = .02), severe anemia (6.1% vs 1.0% vs 1.5%; *P* < .001), and severe rash (2.8% vs 1.2% vs 0.4%; *P* = .047), but a lower incidence of fatigue (44.1% vs 46.9% vs 55.6%; *P* = .005), neutropenia (38.5% vs 39.7% vs 49.8%; *P* = .002), severe diarrhea (6.1% vs 9.1% vs 13.0%; *P* = .02), severe fatigue (4.5% vs 5.5% vs 9.5%; *P* = .02), and severe neutropenia (25.7% vs 26.5% vs 38.1%; *P* < .001). Patients younger than 50 years had an earlier onset of nausea and vomiting (1.0 vs 2.1 vs 2.6 weeks; *P* = .01), mucositis (3.6 vs 5.1 vs 5.7 weeks; *P* = .05), and neutropenia (8.0 vs 9.4 vs 8.4 weeks; *P* = .04), as shown in Figure 2C and eTable 4 in Supplement 1. In addition, patients younger than 50 years had a shorter duration of mucositis (0.6 vs 0.9 vs 1.0 weeks; *P* = .006) as shown in Figure 2D and eTable 5 in Supplement 1.

### Association of Treatment-Related Adverse Events With OS and PFS

**Figure 3.**
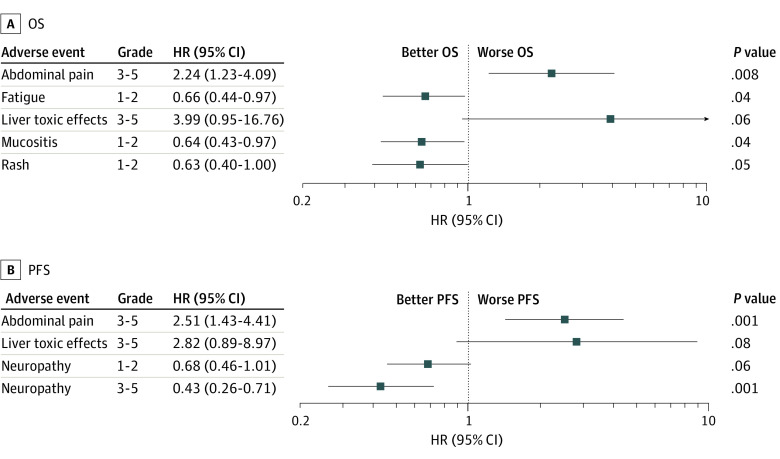
Association of Adverse Events With Overall Survival (OS) and Progression-Free Survival (PFS) in Patients With Metastatic Colorectal Cancer From Study 1 and Study 2 HR indicates hazard ratio.

As summarized in Figure 3A and eTable 6 in Supplement 1, severe abdominal pain (HR, 2.24; 95% CI, 1.23-4.09; *P* = .008) and severe liver toxic effects (HR, 3.99; 95% CI, 0.95-16.76; *P* = .06) were associated with worse OS. In contrast, moderate (grade 1/2) fatigue (HR, 0.66; 95% CI, 0.44-0.97; *P* = .008), moderate mucositis (HR, 0.64; 95% CI, 0.43-0.97; *P* = .04), and moderate rash (HR, 0.63; 95% CI, 0.40-1.00; *P* = .049) were associated with better OS. Similarly, severe abdominal pain (HR, 2.51; 95% CI, 1.43-4.41; *P* = .001) and severe liver toxic effects (HR, 2.82; 95% CI, 0.89-8.97; *P* = .08) were associated with poor PFS. In contrast, severe peripheral neuropathy (HR, 0.43; 95% CI, 0.26-0.71; *P* = .001) was associated with better PFS (Figure 3B; eTable 6 in Supplement 1).

### Survival Outcome and Genetic Alterations

**Figure 4.**
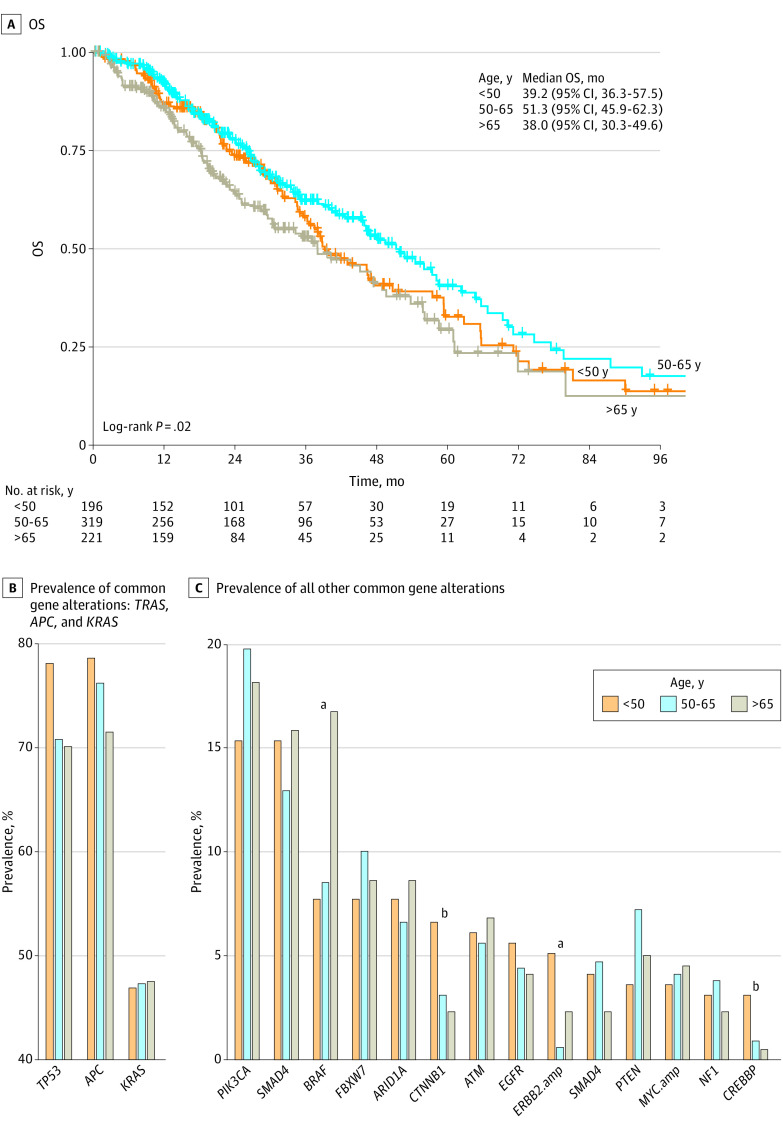
Age-Related Disparity in Survival and Gene Mutations Among Patients With Metastatic Colorectal Cancer From the Moffitt Cancer Center Cohort OS indicates overall survival. ^a^*P* < .01. ^b^*P* < .05.

As reported in eTable 7 in Supplement 1, of the 736 patients in the MCC cohort, 196 (26.6%) were younger than 50 years, 319 (43.3%) were 50 to 65 years, and 221 (30.0%) were older than 65 years. Patients younger than 50 years were more likely to receive triplet therapy and carry a left-sided tumor, while those older than 65 years had a higher rate of patients who were White. Despite the difference of baseline characteristics between the MCC cohort and study 1 and 2 (Table), patients younger than 50 years consistently showed worse median OS compared with those aged 50 to 65 years and comparable median OS to those older than 65 years (39.2 vs 51.3 vs 38.0 months; *P* = .02) (Figure 4A). Further examination of genetic alterations was performed to explore the potential underlying factors in this age-related disparity of survival outcome in patients with mCRC. As presented in Figure 4B and eTable 8 in Supplement 1, we found that all 3 age groups had very similar genetic alterations except that, compared with the other 2 age groups, the tumors of patients younger than 50 years had a higher prevalence of *CTNNB1* mutation (6.6% vs 3.1% vs 2.3%; *P* = .047), *ERBB2* amplification (5.1% vs 0.6% vs 2.3%; *P* = .005), and *CREBBP* mutation (3.1% vs 0.9% vs 0.5%; *P* = .05). Both patients younger than 50 years and those aged 50 to 65 years exhibited a lower incidence of *BRAF* mutation than those older than 65 years (7.7% vs 8.5% vs 16.7%; *P* = .002).

## Discussion

A hypothesis for an increased incidence of EO-CRC despite a decrease in the overall incidence^5,6^ was that EO-CRC may be a different disease as reflected by distinct clinicopathologic and molecular features.^5,39^ Previous studies that attempted to investigate such age-related disparity of survival outcomes had discordant findings. Several studies found an association between younger age and worse survival,^25,29,40,41^ whereas other studies revealed opposite findings.^24,26,27,30,42,43^ Lieu and colleagues^29^ observed younger and older ages were associated with worse survival in patients with mCRC. However, other studies showed at least comparable^26,27,28,30,31^ if not better prognosis^44^ in patients with EO-mCRC. To our knowledge, the present study is the first analysis of individual patient data from clinical trials and a more contemporary cohort that observed worse survival outcomes in patients with EO-mCRC. The inclusion of an external validation cohort from a different time period and with diverse baseline characteristics may have helped to enhance the reliability of our findings.

Inconsistent findings from some of the previous studies could be partially attributed to the dichotomization of patient populations with a single cutoff at age 50 years. The assumption of homogeneity of patients older than 50 years was not valid, as an abundance of literature in geriatric oncology has reported unique treatment response and outcome in patients who are very old.^45,46^ For example, in the study of Lieu and colleagues,^29^ when age was treated as a continuous variable, older age (age >65 years) was associated with worse survival, presumably due to reduced life expectancy, increased comorbidities, or inability to tolerate aggressive chemotherapy.^47^ Therefore, in our study, we divided patients into 3 age groups (<50, 50-65, and >65 years). As expected, we found that patients both younger than 50 and those older than 65 years had worse OS compared with those aged 50 to 65 years. Another distinct feature of our study is that patients received the same treatment, first-line FOLFOX, to minimize possible risk modification from different treatment regimens on survival. Similar observations have been found in our contemporary patient cohort who received different treatment regimens, including triplet chemotherapy with biologic agents.

Differences in baseline characteristics could also prevent us from observing the age-related disparity of survival outcomes in previous studies. In our study, we found a higher proportion of women in patients with EO-mCRC in line with previous studies,^24,27,28,42,48^ likely due to selection bias or differential genetic susceptibility. Meanwhile, consistent with previous studies,^26,48^ a lower proportion of White patients was observed in the EO-mCRC group. Given prior studies that suggested sex and race disparity on survival,^49^ we performed multivariable analysis to adjust for sex, race, and ECOG performance status, which was better at baseline in younger patients.^26^

To use excellent recording of treatment-related adverse events in clinical trial data, we also compared these data among the 3 age groups and observed age-related disparity in patients with mCRC. Consistent with previous findings in the adjuvant setting,^24,25,26,27,28^ our data showed an increased incidence of nausea and vomiting but a decreased incidence of diarrhea, fatigue, and neutropenia in younger patients. The previously reported potential improvement of the toxic effect profile in younger patients^27,50^ was not entirely supported by our study, as we observed a higher incidence of severe abdominal pain, severe anemia, and severe rash in patients with EO-mCRC. These are in line with the findings from the IDEA trials, which revealed an increased incidence of severe nausea and vomiting in younger patients.^25^ In addition, EO-mCRC had an earlier onset of nausea and vomiting, mucositis, and neutropenia. Hence, it is more appropriate to say that patients with EO-mCRC exhibited a unique pattern of adverse events after receiving first-line FOLFOX therapy. Moreover, the association between severe abdominal pain and severe liver toxic effects with worse survival in younger patients suggested an individualized approach to the monitoring and management of these unique treatment-related adverse events.

The age-related disparity of survival and adverse events may suggest unique underlying disease biologic factors in different age groups. Comprehensive genomic profiling in some studies noted a higher prevalence of *TP53* and *CTNNB1* mutations, but a lower prevalence of *BRAF* and *APC* mutations in EO-mCRC,^11,28,43,51,52^ which is consistent with some findings in our study.

The *CTNNB1* and *CREBBP* genes are involved in the Wnt/β-catenin pathway,^53^ and *ERBB2* amplification is involved in the MAPK/ERK pathway.^54^ Thus, our study suggests that these pathways might be implicated in worse survival outcomes in EO-mCRC. However, it is still unclear whether these differential genetic alterations might partially explain the observed disparity in survival outcome given the inconsistent findings across studies and the numerically small differences in few of many genes. Therefore, further efforts should likely focus on multiomic studies to inform disease biologic factors and therapy.

### Limitations

This study has limitations. The 3 clinical trials took place in an era during which biologic agents had not become standard first-line therapy for patients with mCRC and clinical NGS was not used to guide treatment. These clinical trials were conducted with FOLFOX treatment before more intensive therapy, such as folinic acid, fluorouracil, oxaliplatin and irinotecan, became available. Furthermore, they did not collect information on treatment intensity, adherence, and location and number of metastases, which prevented us from adjusting for these factors in our analysis. To compensate and address these limitations intrinsic to the data used in our study, we incorporated institutional patient and clinical NGS data from the modern era, encompassing a more diverse range of patients and treatments, as an external validation cohort. Distributions of some important baseline characteristics, such as race and ethnicity, tumor sidedness, and first-line treatments in this MCC patient cohort were consistent with those previously reported.^26,28,31,48^ However, we acknowledge a limitation in not evaluating adverse events, PFS, or conducting multivariable survival analysis in this cohort due to the following concerns: retrospective data collection, even when sourced from prospectively maintained databases, is susceptible to selection bias and inaccuracy; and the highly heterogeneous nature of this patient population concerning multiple genes, treatments, and histopathologic characteristics may lead to overfitting and nonconvergence of the multivariable model, given the relatively small sample size of this patient cohort. Consequently, we used Moffitt data solely for external validation of OS, rather than for the primary analysis. In addition, these findings may not generalize to other racial and ethnic categories given that the majority of trial participants were identified as White individuals.

## Conclusions

In this cohort study, patients with EO-mCRC who received first-line treatment appeared to have worse survival compared with their older counterparts and experienced unique treatment-related adverse events. Distinct genomic profiles could possibly explain the disparities in EO-mCRC. These findings might improve an individualized approach to chemotherapy, counseling, and management of treatment-related adverse events in this patient population.

## Notes

Supplement 1. **eTable 1.** Demographic and Clinical Characteristics of Patients With Metastatic Colorectal Cancer From Study 1 and Study 2 Stratified by Age Groups **eTable 2.** The Prognostic Values of Age Groups and Other Common Demographic and Clinical Factors in Patients With Metastatic Colorectal Cancer From Study 1 and Study 2 **eTable 3.** Incidence (%) of Adverse Events for Each Age Group From Study 1 and Study 2 **eTable 4.** Median (IQR) Time to Onset (Weeks) of Adverse Events for Each Age Group From Study 1 and Study 2 **eTable 5.** Median (IQR) Time to Resolution (Weeks) of Adverse Events for Each Age Group From Study 1 and Study 2 **eTable 6.** The Prognostic Value of Adverse Events (None vs Grade 1-2 vs Grade 3-5) in Patients With Early-Onset Metastatic Colorectal Cancer From Study 1 and Study 2 **eTable 7.** Demographic and Clinical Characteristics of Patients With Metastatic Colorectal Cancer in the Moffitt Cancer Center Cohort Stratified by Age Groups **eTable 8.** Prevalence of Common Gene Mutations in Patients With Metastatic Colorectal Cancer From the Moffitt Cancer Center Cohort Stratified by Age Groups **eMethods.** Study Design and Methods of Three Clinical Trials Evaluating First-Line Treatment for Metastatic Colorectal Cancer: NCT00272051, NCT00305188, and NCT0036401

Supplement 2.
Data Sharing Statement
