# Supplementary material for: Association of Age With Treatment-Related Adverse Events and Survival in Patients With Metastatic Colorectal Cancer
Source: JAMA Netw Open. 2023 Jun 26;6(6):e2320035. doi: 10.1001/jamanetworkopen.2023.20035 (PMC10293914; doi:10.1001/jamanetworkopen.2023.20035)
Supplement: Supplement 1. — eTable 1. Demographic and Clinical Characteristics of Patients With Metastatic Colorectal Cancer From Study 1 and Study 2 Stratified by Age Groups eTable 2. The Prognostic Values of Age Groups and Other Common Demographic and Clinical Factors in Patients With Metastatic Colorectal Cancer From Study 1 and Study 2 eTable 3. Incidence (%) of Adverse Events for Each Age Group From Study 1 and Study 2 eTable 4. Median (IQR) Time to Onset (Weeks) of Adverse Events for Each Age Group From Study 1 and Study 2 eTable 5. Median (IQR) Time to Resolution (Weeks) of Adverse Events for Each Age Group From Study 1 and Study 2 eTable 6. The Prognostic Value of Adverse Events (None vs Grade 1-2 vs Grade 3-5) in Patients With Early-Onset Metastatic Colorectal Cancer From Study 1 and Study 2 eTable 7. Demographic and Clinical Characteristics of Patients With Metastatic Colorectal Cancer in the Moffitt Cancer Center Cohort Stratified by Age Groups eTable 8. Prevalence of Common Gene Mutations in Patients With Metastatic Colorectal Cancer From the Moffitt Cancer Center Cohort Stratified by Age Groups eMethods. Study Design and Methods of Three Clinical Trials Evaluating First-Line Treatment for Metastatic Colorectal Cancer: NCT00272051, NCT00305188, and NCT0036401 [file jamanetwopen-e2320035-s001.pdf]

Supplementary Online Content

Meng L, Thapa R, Delgado MG, et al. Association of age with treatment-related adverse events and survival in patients with metastatic colorectal cancer. *JAMA Netw Open*. 2023;6(6):e2320035. doi:10.1001/jamanetworkopen.2023.20035

eTable 1. Demographic and Clinical Characteristics of Patients With Metastatic Colorectal Cancer From Study 1 and Study 2 Stratified by Age Groups

eTable 2. The Prognostic Values of Age Groups and Other Common Demographic and Clinical Factors in Patients With Metastatic Colorectal Cancer From Study 1 and Study 2

eTable 3. Incidence (%) of Adverse Events for Each Age Group From Study 1 and Study 2

eTable 4. Median (IQR) Time to Onset (Weeks) of Adverse Events for Each Age Group From Study 1 and Study 2

eTable 5. Median (IQR) Time to Resolution (Weeks) of Adverse Events for Each Age Group From Study 1 and Study 2

eTable 6. The Prognostic Value of Adverse Events (None vs Grade 1-2 vs Grade 3-5) in Patients With Early-Onset Metastatic Colorectal Cancer From Study 1 and Study 2

eTable 7. Demographic and Clinical Characteristics of Patients With Metastatic Colorectal Cancer in the Moffitt Cancer Center Cohort Stratified by Age Groups

eTable 8. Prevalence of Common Gene Mutations in Patients With Metastatic Colorectal Cancer From the Moffitt Cancer Center Cohort Stratified by Age Groups

eMethods. Study Design and Methods of Three Clinical Trials Evaluating First-Line Treatment for Metastatic Colorectal Cancer: NCT00272051, NCT00305188, and NCT0036401

This supplementary material has been provided by the authors to give readers additional information about their work.

© 2023 Meng L et al. *JAMA Network Open.*

eTable 1. Demographic and clinical characteristics of patients with metastatic colorectal cancer from Study 1 and Study 2 stratified by age groups.

	< 50 (n=179)	50 - 65 (n=582)	> 65 (n=462)	P value	Overall (n=1223)
Gender, n (%)				<0.001	
Female	98 (54.7)	226 (38.8)	162 (35.1)		486 (39.7)
Male	81 (45.3)	356 (61.2)	300 (64.9)		737 (60.3)
Race, n (%)				0.012	
White	158 (88.3)	551 (94.7)	431 (93.3)		1140 (93.2)
Other	21 (11.7)	31 (5.3)	31 (6.7)		83 (6.8)
ECOG, n (%)				0.32	
0/1	175 (98.3)	565 (97.1)	442 (96.1)		1182 (96.9)
2	3 (1.7)	17 (2.9)	18 (3.9)		38 (3.1)

© 2023 Meng L et al. *JAMA Network Open.*

eTable 2. The prognostic values of age groups and other common demographic and clinical factors in patients with metastatic colorectal cancer from Study 1 and Study 2.

Overall survival					
	Variable	Univariate		Multivariable	
		HR (95%CI)	P value	HR (95%CI)	P value
Age	<50 vs 50-65	1.50 (1.21 - 1.85)	<0.001	1.48 (1.19 - 1.84)	<0.001
	>65 vs 50-65	1.17 (1.00 - 1.38)	0.053	1.15 (0.97 - 1.35)	0.10
Gender	Male vs Female	0.98 (0.84 - 1.14)	0.82	1.00 (0.86 - 1.17)	0.99
Race	Other vs White	1.19 (0.87 - 1.62)	0.27	1.09 (0.80 - 1.49)	0.57
ECOG	2 vs 0/1	2.49 (1.75, 3.54)	<0.001	2.41 (1.69 - 3.44)	<0.001

Progression-free survival					
	Variable	Univariate		Multivariable	
		HR (95%CI)	P value	HR (95%CI)	P value
Age	<50 vs 50-65	1.46 (1.22 - 1.75)	<0.001	1.46 (1.22 - 1.76)	<0.001
	>65 vs 50-65	1.10 (0.97 - 1.26)	0.15	1.10 (0.96 - 1.26)	0.17
Gender	Male vs Female	0.98 (0.87 - 1.11)	0.78	1.01 (0.89 - 1.14)	0.92
Race	Other vs White	0.95 (0.74 - 1.22)	0.69	0.89 (0.69 - 1.14)	0.37
ECOG	2 vs 0/1	1.54 (1.10 - 2.15)	0.011	1.55 (1.11 - 2.17)	0.010

© 2023 Meng L et al. *JAMA Network Open.*

eTable 3. Incidence (%) of adverse events for each age group from Study 1 and Study 2.

AE	All grade				Grade 3, 4, & 5			
	Age <50	Age 50-65	Age >65	P	Age <50	Age 50-65	Age >65	P
Abdominal pain	71 (39.7)	180 (30.9)	145 (31.4)	0.082	15 (8.4)	20 (3.4)	16 (3.5)	0.018
Anemia	23 (12.8)	53 (9.1)	45 (9.7)	0.334	11 (6.1)	6 (1.0)	7 (1.5)	<0.001
Chest pain	5 (2.8)	21 (3.6)	12 (2.6)	0.656	0 (0.0)	1 (0.2)	1 (0.2)	0.99
Diarrhea	92 (51.4)	281 (48.3)	264 (57.1)	0.017	11 (6.1)	53 (9.1)	60 (13.0)	0.02
DVT/PE	8 (4.5)	33 (5.7)	35 (7.6)	0.286	4 (2.2)	23 (4.0)	26 (5.6)	0.152
Fatigue	79 (44.1)	273 (46.9)	257 (55.6)	0.0052	8 (4.5)	32 (5.5)	44 (9.5)	0.019
Liver toxicity	9 (5.0)	31 (5.3)	14 (3.0)	0.179	3 (1.7)	8 (1.4)	8 (1.7)	0.858
Mucositis	66 (36.9)	183 (31.4)	164 (35.5)	0.242	4 (2.2)	6 (1.0)	15 (3.2)	0.034
Nausea/vomiting	124 (69.3)	335 (57.6)	279 (60.4)	0.019	5 (2.8)	20 (3.4)	23 (5.0)	0.346
Neuropathy	153 (85.5)	494 (84.9)	396 (85.7)	0.932	32 (17.9)	99 (17.0)	84 (18.2)	0.881
Neutropenia	69 (38.5)	231 (39.7)	230 (49.8)	0.0018	46 (25.7)	154 (26.5)	176 (38.1)	<0.001
Rash	52 (29.1)	143 (24.6)	104 (22.5)	0.224	5 (2.8)	7 (1.2)	2 (0.4)	0.047
Thrombocytopenia	31 (17.3)	123 (21.1)	117 (25.3)	0.068	4 (2.2)	13 (2.2)	19 (4.1)	0.195

© 2023 Meng L et al. *JAMA Network Open.*

eTable 4. Median (IQR) time to onset (weeks) of adverse events for each age group from Study 1 and Study 2. IQR, interquartile range.

AE	Age <50<br>n = 141	Age 50-65<br>n = 446	Age >65<br>n = 362	P
Fatigue	4.1 (1.3;11.6)	5.4 (1.8;14.1)	4.6 (1.0;14.4)	0.356
Nausea/vomiting	1.0 (0.4;5.0)	2.1 (0.4;6.4)	2.6 (0.6;9.7)	0.012
Diarrhea	4.3 (1.6;11.6)	4.6 (1.7;11.9)	3.4 (0.9;10.0)	0.044
Mucositis	3.6 (0.9;8.8)	5.1 (2.0;12.1)	5.7 (2.1;13.9)	0.051
Neuropathy	4.3 (0.9;12.3)	4.8 (2.1;14.4)	4.6 (2.0;14.4)	0.697
Fatigue	4.1 (1.3;11.6)	5.4 (1.8;14.1)	4.6 (1.0;14.4)	0.356
Neutropenia	8.0 (4.0;14.4)	9.4 (6.1;15.2)	8.4 (4.1;14.1)	0.043
Thrombocytopenia	12.6 (7.1;17.9)	12.4 (7.9;18.5)	12.0 (6.3;21.1)	0.993
Chest pain	8.1 (2.3;13.6)	18.0 (7.1;30.4)	12.0 (4.5;16.5)	0.195
DVT/PE	14.1 (7.3;23.2)	8.1 (4.3;15.3)	8.1 (5.4;14.6)	0.366
Anemia	4.7 (2.2;15.3)	8.1 (4.1;16.6)	10.1 (2.6;20.1)	0.535
Liver toxicity	10.1 (8.3;17.3)	19.5 (6.1;25.8)	8.5 (7.0;16.5)	0.521

© 2023 Meng L et al. *JAMA Network Open.*

eTable 5. Median (IQR) time to resolution (weeks) of adverse events for each age group from Study 1 and Study 2. IQR, interquartile range.

AE	Age <50<br>n = 112	Age 50-65<br>n = 305	Age >65<br>n = 246	P
Fatigue	1.0 (0.4;2.0)	0.9 (0.3;2.7)	1.0 (0.3;2.4)	0.931
Nausea/vomiting	0.3 (0.1;0.6)	0.3 (0.0;0.6)	0.3 (0.1;0.7)	0.240
Diarrhea	0.3 (0.1;0.6)	0.3 (0.1;0.7)	0.3 (0.1;0.7)	0.642
Mucositis	0.6 (0.3;1.0)	0.9 (0.3;1.9)	1.0 (0.6;1.6)	0.006
Neuropathy	0.6 (0.3;2.0)	0.7 (0.3;1.9)	0.7 (0.4;1.7)	0.937
Fatigue	1.0 (0.4;2.0)	0.9 (0.3;2.7)	1.0 (0.3;2.4)	0.931
Neutropenia	1.0 (0.9;1.9)	1.0 (0.9;1.4)	1.0 (1.0;1.9)	0.060
Thrombocytopenia	1.1 (0.9;2.1)	1.0 (1.0;2.2)	1.0 (1.0;2.9)	0.850
Chest pain	0.6 (0.0;6.6)	0.2 (0.0;0.4)	1.9 (0.0;3.7)	0.401
DVT/PE	1.3 (0.4;4.7)	6.6 (2.0;14.2)	5.7 (0.9;11.1)	0.379
Anemia	3.3 (0.6;5.2)	2.0 (0.9;5.0)	1.3 (0.2;3.8)	0.471
Liver toxicity	1.6 (1.0;2.2)	2.1 (1.2;4.3)	2.0 (1.6;2.7)	0.483

© 2023 Meng L et al. *JAMA Network Open.*

eTable 6. The prognostic value of adverse events (none vs grade 1-2 vs grade 3-5) in patients with early-onset metastatic colorectal cancer from Study 1 and Study 2.

AE	Grade	Overall Survival			Progression Free Survival		
		HR (95% CI)	P	Overall P	HR (95% CI)	P	Overall P
Abdominal pain	1-2	1.46 (0.98, 2.18)	0.066	0.015	1.19 (0.84, 1.68)	0.323	0.006
	3-5	2.24 (1.23, 4.09)	0.008		2.51 (1.43, 4.41)	0.001	
Anaemia	1-2	1.34 (0.68, 2.65)	0.401	0.664	1.30 (0.72, 2.36)	0.381	0.682
	3-5	1.17 (0.54, 2.52)	0.692		1.03 (0.52, 2.04)	0.924	
Chest pain	1-2	2.33 (0.85, 6.34)	0.099		1.50 (0.61, 3.67)	0.375	
Diarrhoea	1-2	0.83 (0.57, 1.21)	0.342	0.528	0.83 (0.60, 1.15)	0.259	0.528
	3-5	1.19 (0.51, 2.77)	0.690		0.91 (0.47, 1.76)	0.773	
DVT/PE	1-2	1.06 (0.33, 3.34)	0.927	0.986	1.58 (0.58, 4.29)	0.370	0.419
	3-5	1.09 (0.34, 3.43)	0.886		0.62 (0.23, 1.67)	0.343	
Fatigue	1-2	0.66 (0.44, 0.97)	0.035	0.099	1.01 (0.72, 1.39)	0.976	0.969
	3-5	0.71 (0.31, 1.64)	0.421		1.10 (0.53, 2.27)	0.801	
Liver toxicity	1-2	0.62 (0.23, 1.68)	0.344	0.104	0.59 (0.24, 1.44)	0.247	0.104
	3-5	3.99 (0.95, 16.76)	0.059		2.82 (0.89, 8.97)	0.079	
Mucositis	1-2	0.64 (0.43, 0.97)	0.035	0.098	0.90 (0.64, 1.25)	0.523	0.588
	3-5	1.13 (0.35, 3.57)	0.841		1.45 (0.53, 3.96)	0.466	
Nausea/vomiting	1-2	0.98 (0.66, 1.45)	0.902	0.597	0.91 (0.64, 1.28)	0.587	0.262
	3-5	1.78 (0.54, 5.82)	0.340		1.91 (0.75, 4.86)	0.172	
Neuropathy	1-2	0.85 (0.54, 1.34)	0.484	0.251	0.68 (0.46, 1.01)	0.055	0.005
	3-5	0.60 (0.32, 1.10)	0.100		0.43 (0.26, 0.71)	0.001	
Neutropenia	1-2	0.93 (0.53, 1.62)	0.792	0.884	0.97 (0.60, 1.57)	0.902	0.743
	3-5	0.90 (0.59, 1.38)	0.638		0.87 (0.60, 1.25)	0.442	
Rash	1-2	0.63 (0.40, 1.00)	0.049	0.141	0.83 (0.57, 1.19)	0.304	0.590
	3-5	0.81 (0.26, 2.57)	0.723		0.94 (0.38, 2.31)	0.891	
Thrombocytopenia	1-2	0.83 (0.49, 1.41)	0.488	0.671	1.15 (0.74, 1.79)	0.522	0.572
	3-5	1.30 (0.48, 3.55)	0.610		1.57 (0.58, 4.26)	0.377	

© 2023 Meng L et al. *JAMA Network Open.*

eTable 7. Demographic and clinical characteristics of patients with metastatic colorectal cancer in the Moffitt Cancer Center cohort stratified by age groups.

	**< 50** (n=196)	**50 - 65** (n=319)	**> 65** (n=221)	**P value**	**Overall** (n=736)
**Gender**, n (%)				0.488	
Female	88 (44.9)	135 (42.3)	105 (47.5)		328 (44.6)
Male	108 (55.1)	184 (57.7)	116 (52.5)		408 (55.4)
**Race**, n (%)				0.044	
White	159 (81.1)	255 (79.9)	188 (85.5)		602 (81.8)
Other	37 (18.9)	64 (20.1)	33 (14.5)		134 (18.2)
**First line therapy**, n (%)				<0.001	
Single	12 (6.1)	17 (5.3)	32 (14.5)		61 (8.3)
Doublet	157 (80.1)	283 (88.7)	184 (83.3)		624 (84.8)
Triplet	27 (13.8)	19 (6.0)	5 (2.3)		51 (6.9)
**Sidedness**, n (%)					
Left	142 (76.3)	221 (71.1)	118 (57.6)	<0.001	481 (68.5)
Right	44 (23.7)	90 (28.9)	87 (42.4)		221 (31.5)
**MSI status**, n (%)				0.286	
MSI	8 (4.4)	16 (5.7)	16 (8.2)		40 (6.1)
MSS	172 (95.6)	265 (94.3)	178 (91.8)		615 (93.9)
**TMB level**, n (%)				0.764	
Low (< 10)	62 (88.6)	102 (85.7)	64 (88.9)		228 (87.4)
High (≥ 10)	8 (11.4)	17 (14.3)	8 (11.1)		33 (12.6)

© 2023 Meng L et al. *JAMA Network Open.*

eTable 8. Prevalence of common gene mutations in patients with metastatic colorectal cancer from the Moffitt Cancer Center cohort stratified by age groups.

**Gene**	**<50** (n=196)<br>n (%)	**50-65** (n=319)<br>n (%)	**>65** (n=221)<br>n (%)	**P Value**
TP53	153 (78.1)	226 (70.8)	155 (70.1)	0.13
APC	154 (78.6)	243 (76.2)	158 (71.5)	0.22
KRAS	92 (46.9)	151 (47.3)	105 (47.5)	0.99
PIK3CA	30 (15.3)	63 (19.7)	40 (18.1)	0.44
SMAD4	30 (15.3)	41 (12.9)	35 (15.8)	0.57
BRAF	15 (7.7)	27 (8.5)	37 (16.7)	0.002
FBXW7	15 (7.7)	32 (10.0)	19 (8.6)	0.64
ARID1A	15 (7.7)	21 (6.6)	19 (8.6)	0.68
CTNNB1	13 (6.6)	10 (3.1)	5 (2.3)	0.047
ATM	12 (6.1)	18 (5.6)	15 (6.8)	0.86
EGFR	11 (5.6)	14 (4.4)	9 (4.1)	0.73
*ERBB2.*amp	10 (5.1)	2 (0.6)	5 (2.3)	0.005
NRAS	8 (4.1)	15 (4.7)	5 (2.3)	0.34
PTEN	7 (3.6)	23 (7.2)	11 (5.0)	0.20
*MYC.*amp	7 (3.6)	13 (4.1)	10 (4.5)	0.89
NF1	6 (3.1)	12 (3.8)	5 (2.3)	0.62
CREBBP	6 (3.1)	3 (0.9)	1 (0.5)	0.050

© 2023 Meng L et al. *JAMA Network Open.*

eMethods. Study design and methods of three clinical trials evaluating first-line treatment for metastatic colorectal cancer: NCT00272051, NCT00305188, and NCT00364013.

The three clinical trials included in this study were NCT00272051, NCT00305188, and NCT00364013. All three trials had a randomized, open-label, multicenter design, and included patients with metastatic colorectal cancer (mCRC) who had not received prior chemotherapy for metastatic disease. The inclusion criteria were similar across all trials, including age ⩾18 years, histologically confirmed mCRC, Eastern Cooperative Oncology Group (ECOG) performance status ⩽2, and adequate hematologic, hepatic, and renal function. Patients with a history of brain metastases, uncontrolled cardiovascular disease, or another primary cancer were excluded.

The interventions in all trials involved the administration of 5-fluorouracil and oxaliplatin (FOLFOX) as first-line treatment for mCRC. In the NCT00272051 trial, patients were randomized to receive either FOLFOX with xaliproden or FOLFOX with placebo to evaluate the efficacy of xaliproden in reducing neurotoxicity. The NCT00305188 trial evaluated the efficacy of xaliproden in preventing neurotoxicity in patients receiving FOLFOX. The NCT00364013 trial evaluated the safety and efficacy of FOLFOX in combination with panitumumab in patients with mCRC.

The primary endpoint of all trials was overall survival (OS), while secondary endpoints included progression-free survival (PFS), response rate, and adverse events (AEs). The primary AE of interest was treatment-related toxicity, including grade 3 or higher diarrhea, nausea, vomiting, and neuropathy.

In summary, the three clinical trials included in this study had a randomized, open-label, multicenter design and evaluated the efficacy and safety of FOLFOX as first-line treatment for mCRC. The primary endpoint was overall survival, and the primary adverse events of interest were treatment-related toxicities.

© 2023 Meng L et al. *JAMA Network Open.*
