# Supplementary material for: Association of Age With Treatment-Related Adverse Events and Survival in Patients With Metastatic Colorectal Cancer
Source: JAMA Netw Open. 2023 Jun 26;6(6):e2320035. doi: 10.1001/jamanetworkopen.2023.20035 (PMC10293914; doi:10.1001/jamanetworkopen.2023.20035)
Supplement: Supplement 2. — Data Sharing Statement [file jamanetwopen-e2320035-s002.pdf]

Data Sharing Statement

Meng. Association of Age With Treatment-Related Adverse Events and Survival in Patients With Metastatic Colorectal Cancer. *JAMA Netw Open*. Published June 26, 2023.
doi:10.1001/jamanetworkopen.2023.20035

Data
Data available: Yes
Data types: Deidentified participant data
How to access data: https://data.projectdatasphere.org/projectdatasphere/html/content/136
https://data.projectdatasphere.org/projectdatasphere/html/content/137
When available: With publication

Supporting Documents
Document types: Other (please specify)
Additional Information: previous version submitted to medRxiv
How to access documents: https://www.medrxiv.org/content/10.1101/2022.10.08.22280865v1
When available: With publication

Additional Information
Who can access the data: anyone requesting the data
Types of analyses: for any purpose
Mechanisms of data availability: without investigator support
